# Morphological and Ultrastructural Collagen Defects: Impact and Implications in Dentinogenesis Imperfecta

**DOI:** 10.3390/dj11040095

**Published:** 2023-04-03

**Authors:** Lubabah S. A. Gadi, David Y. S. Chau, Susan Parekh

**Affiliations:** 1 Department of Paediatric Dentistry, Eastman Dental Institute, University College London, Bloomsbury Campus, Rockefeller Building, 21 University Street, London WC1E 6DE, UKs.parekh@ucl.ac.uk (S.P.); 2 Department of Paediatric Dentistry, King Abdulaziz University Dental Hospital, Al Ehtifalat Street, Jeddah 22252, Saudi Arabia; 3 Department of Division of Biomaterials and Tissue Engineering, Eastman Dental Institute, University College London, Royal Free Campus, Rowland Hill Street, London NW3 2PF, UK

**Keywords:** collagen, D-banding, demineralisation, dentin, fibril, osteogenesis imperfecta

## Abstract

Collagen is the building block for the extracellular matrix in bone, teeth and other fibrous tissues. Osteogenesis imperfecta (OI), or brittle bone disease, is a heritable disorder that results from defective collagen synthesis or metabolism, resulting in bone fragility. The dental manifestation of OI is dentinogenesis imperfecta (DI), a genetic disorder that affects dentin structure and clinical appearance, with a characteristic feature of greyish-brown discolouration. The aim of this study was to conduct a systematic review to identify and/or define any ultrastructural changes in dentinal collagen in DI. Established databases were searched: Cochrane Library, OVID Embase, OVID Medline and PubMed/Medline. Search strategies included: Collagen Ultrastructure, DI and OI. Inclusion criteria were studies written in English, published after 1990, that examined human dental collagen of teeth affected by DI. A Cochrane data extraction form was modified and used for data collection. The final dataset included seventeen studies published from 1993 to 2021. The most prevalent findings on collagen in DI teeth were increased coarse collagen fibres and decreased fibre quantity. Additional findings included changes to fibre orientation (i.e., random to parallel) and differences to the fibre organisation (i.e., regular to irregular). Ultrastructural defects and anomalies included uncoiled collagen fibres and increased D-banding periodicity. Studies in collagen structure in DI reported changes to the surface topography, quantity, organisation and orientation of the fibres. Moreover, ultrastructural defects such as the packing/coiling and D-banding of the fibrils, as well as differences in the presence of other collagens are also noted. Taken together, this study provides an understanding of the changes in collagen and its impact on clinical translation, paving the way for innovative treatments in dental treatment.

## 1. Introduction

The following work is part of a defended thesis [1]. Collagen type I is the most abundant protein of the extracellular matrix in skin, vessels, heart, lungs and bone [2]. Bone is composed of mineralised collagen, which is 90% collagen type I [3]. Collagen synthesis is a highly regulated biochemical process that involves numerous enzymes and cofactors. An error occurring in any step of collagen synthesis can lead to defective collagen formation. This can result from clinical nutritional deficiency, such as scurvy disease or gene mutations including osteogenesis Imperfecta (OI), Ehlers–Danlos syndrome or EDS [4,5].

Osteogenesis imperfecta (OI) is a genetically inherited connective tissue disease characterised by fragile bone, decreased bone mass and multiple fractures [6]. OI is considered a rare bone disorder; in 1981, the disorder affected 6/100,000 individuals in the UK and 21.8/100,000 in Denmark in 1989 [7]. A more recent study conducted in 2015 found the prevalence of OI to be 7.4/100,000 individuals in Sweden [8].

The most widely used classification for OI was established by Sillence. Colleagues in 1979 distinguished four phenotypes of OI: type I is characterised by mild deformity; type II is a lethal perinatal disorder; type III is a moderate yet progressive form; and a varying type IV ranges from mild to severe phenotypes [9]. The most recent classification of OI identified 18 subtypes [10]. This classification was based on a holistic approach, accounting for the clinical presentation, radiographic features, genetic background, mode of inheritance and histological features [10,11] The eighteen subtypes have been grouped into six categories based on the pathophysiology of the mutation. The first category is defects in collagen synthesis and structure, as shown in Table 1 [6,10].

**Figure 1:**
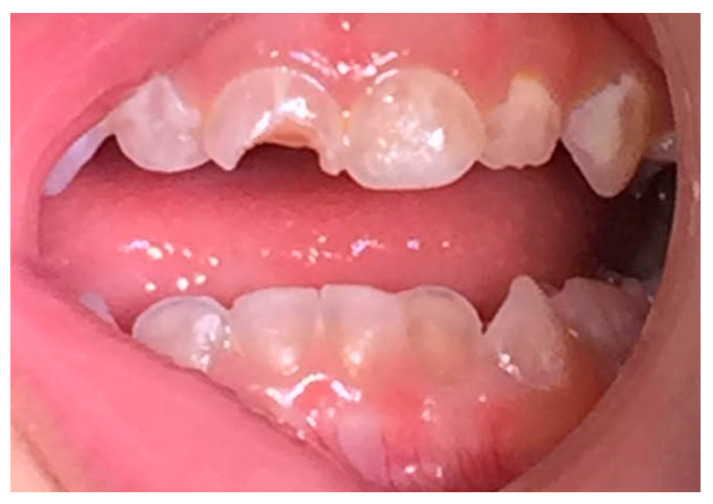
Primary dentition affected by DI demonstrating phenotypic amber hue translucency.

Bone and dentin have many similarities; both are formed by cells that secrete an extracellular matrix, which mainly consists of type I collagen. The fibres form a highly organised fibre scaffold that then undergoes mineralisation. The inorganic component of both is hydroxyapatite in a crystallised form [12]. Given these similarities and because the basis of OI pathophysiology is collagen deformation, teeth can be affected in patients with OI, resulting in dentinogenesis imperfecta (DI) (Figure 1). In OI where the mutation is in type I collagen, DI is considered a greatly penetrant trait [13].

DI, or hereditary opalescent dentin, is a genetic disease in which the mutation affects the dentin structure of either one or both dentitions [14]. The disease presents with characteristic features of greyish-brown discolouration, pulpal obliteration, crown fractures and accelerated tooth wear [15]. DI is considered a rare genetic disorder as it is reported to affect 57/100,000 individuals in France, 90/100,000 in India and as formerly reported by Witkop, 13–17/100,000 of the total population [16,17,18]. 

The disorder can present as part of a syndrome or isolated. The most well-known classification categorises DI into three phenotypes: Syndromic DI or type I, typically associated with OI (OIDI); non-syndromic DI or type II, an isolated form of the disease; and type III is known to be specific to a triracial isolate from Maryland and Washington D.C. [15]. However, the classification was deemed problematic, as the differentiation between Shields types I, II and III was unclear. In 1988, a different classification reported that Shields types II and III were different phenotypes caused by the same type of gene mutations. Therefore, they indicated Shields type I as DI, Shields type II as hereditary opalescent dentin and Shields type III as a Brandywine isolate form of type II, as shown in Table 2 [19]. In 2008, a study by Barron et al. reviewed classification and found that the classification adopted by the Mendelian Inheritance in Man (MIM) abandoned type I DI and only defined types II and III [14]. Accordingly, it necessitated that although Shield’s and Witkop’s classifications were incomprehensive, they were the most valid classifications. In 2015, de La Dure-Molla and colleagues revised the classification of isolated DI and a similar disorder named dentin dysplasia. In their review, it was suggested that types II and III DI are the same condition with variably severity. They denoted type II as moderate DI and type III as severe DI. As for the mild severity, they suggested that type II dentin dysplasia is not a separate condition but rather a mild form of isolated DI [20]. 

Teeth affected by DI clinically can appear normal in shape with an opalescent amber hue, as shown in Figure 1 [21]. and frequently chipped enamel. This clinical presentation is common across all types of DI with variable degree of severity and expression [22]. In both isolated DI and OIDI, primary and permanent dentitions can be affected, with the latter exhibiting milder forms of the disease. Radiographically, teeth in OIDI are reported to have short and constricted roots. Obliteration of the pulp due to dentin hypertrophy is another pathognomonic trait, which can be seen early in developing teeth prior to eruption [14,22]. Similarly, in isolated DI, teeth are characterised by conical roots and pulpal obliteration. However, in this type, bulbous crowns are frequently seen, which can be attributed to the marked constriction near the cervical line [14,16,20]. “Shell teeth” is a term to describe teeth affected by the Brandywine isolated DI. Contrary to the previous two types, teeth of this racial isolate exhibit a marked reduction in dentin structure and pulpal enlargement, hence the prescription. This results in an increased incidence rate in pulp exposure, which is a characteristic finding in type Brandywine isolated DI [14,20,22].

Dentin is known to be a mineralised tissue with collagen forming most of its organic compound. The defect in dentinal collagen defies the breakthroughs in adhesive dentistry. Discovering the damage in dentinal collagen and knowing the ultrastructural defects caused by the disorder can be the first steps to prevent morbid dental complications. An example of this is the invention of a restoration that binds to dentinal collagen rather than micromechanical attachment as most adhesive resin restorations.

Studies on dentin deformity in DI have mainly focused on the macrostructure of collagen. Studies have reported an increased thickness of type I DI collagen diameter [23,24]. Others reported random organisation and disoriented collagen fibres [25,26]. More recent studies found that the defect was in collagen quantity and reported a decrease in collagen fibres [21,27,28]. However, the literature reporting on the ultrastructural dentin collagen defects is limited, especially when compared to studies focusing on dermal or skeletal collagen. Thus, microscopic changes in collagen remain unknown, in addition to how these changes relate to differences in dentin mechanical properties. This review article aims to examine and characterise collagen defects in teeth affected by OIDI and isolated dentinogenesis imperfecta.

## 2. Materials and Methods

### 2.1. Search Strategy and Selection Criteria

Prior to the study implementation, there was no registration of the study protocol in a public database. The chosen databases were OVID Embase, Cochrane Library and PubMed/Medline, in addition to a freehand search using Google Scholar. In June 2020, the databases were searched for ultrastructural collagen defects in teeth with DI and OI. Subject headings and free text words were refined for use in the search concepts by project team members (Supplementary Table S1). Further terms were identified and tested from known relevant papers. The search was reviewed by an information specialist with the aid of UCL library services. The results of the database searches were stored and de-duplicated in an EndNote library. Further relevant studies were sought out by citation searching (forwards and backwards) of the included studies. Search engines used for citation screening included Google Scholar, ResearchGate and PubMed search engines. The numbers of citations for a specific study varied between the search engines, so articles were screened and cross-referenced for relevance. Finally, hand searches on Google Scholar and UCL Explore search engines were additionally carried out.

Inclusion criteria

During the initial stages of article selection, the date of publication was not limited to an interval, nor were articles that examined animal tissues. This was intended to identify the maximum number of studies from the literature and was only adapted when the number of articles obtained was not affected by these criteria. Therefore, the final inclusion criteria applied were:

- Human teeth, both of primary and permanent dentitions, of patients with DI ± OI. Patient age was not limited to a range;
- Teeth must exhibit collagen defects. Although this study focuses on the ultrastructural defects in dental collagen as cross-banding, other defects such as collagen size, shape and density have been included. Teeth could be examined with any assessment method;
- All study designs accepted;
- Publication date 1990 to 2021;
- Studies written in English.

Exclusion criteria:

- Animal studies;
- Non-DI dental pathology: Amelogenesis imperfecta, Dentin dysplasia, dens in dente;
- Studies of OI with no dental collagen examination;
- DI studies with no collagen examination;
- Dental pulp studies and regenerative studies;
- Papers published pre-1990;
- Papers with no English translation.

### 2.2. Data Extraction

A data extraction form (Supplementary Data S1) was developed from the Cochrane Library form, Effective Practice and Organisation of Care (EPOC) Data collection form [29]. Tables of unrelated content, i.e., intervention groups, were deleted. Furthermore, necessary tabs were added for detailed methodology recording. Full search strategy can be found in Supplementary Data S2.

## 3. Results

### 3.1. Study Selection

Database search results were collated and managed in Endnote reference database and were manually checked. Additional grey and clinical trial-related results were profiled in Microsoft Excel (version 2019). At each stage of the elimination process, the number of studies and justification reasoning were recorded. A Preferred Reporting Items for Systematic Reviews and Meta-Analyses (PRISMA) flow diagram methodology was developed. However, a PRISMA checklist was not utilised due to the nature of the study and the specialist support, in-house, of the extraction form. A risk of bias (RoB) assessment for additional parameters (i.e., selection of study, synthesis of data, sources of funding, author’s conflicts of interest statement) was considered but deemed not appropriate as they tend to be designed for meta-analyses—solely for comparisons of randomised control trials (RCT) and generally not ideologically suited for the assessment of non-randomised trials or observational studies. Verification and validation of the form were assessed with an initial set of data by the in-house research team to ensure Q/A consistency throughout the study.

**Figure 2:**
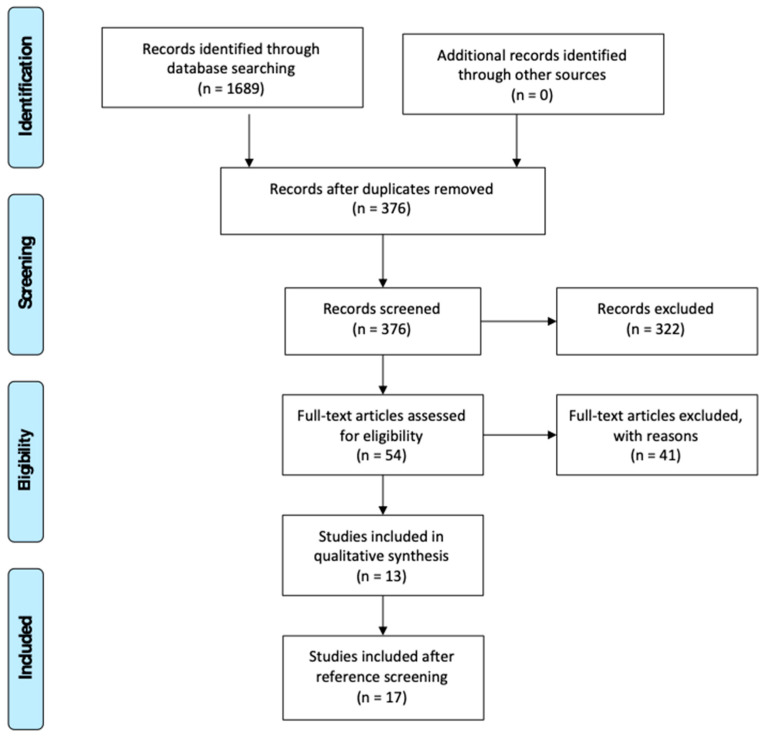
PRISMA flow diagram of article selection process.

The database searches identified 1689 records. Once duplicates were removed, there were 376 records (Figure 2). The identified 376 papers were screened for exclusion by title. 195 studies were excluded. Then, 181 papers were screened by abstract, of which 55 were included. Sixteen non-English articles that did not have a translated version were excluded at this stage.

Full articles of fifty-five studies were retrieved for the final stage of screening. At this stage, publication date was added as a new exclusion criterion. All studies published before the 1990s were excluded and included studies totalled at 13. Finally, using forward and backward citation screening, four studies were found to fulfil the inclusion criteria and were added to the final dataset, reaching 17 articles.

**Figure 3:**
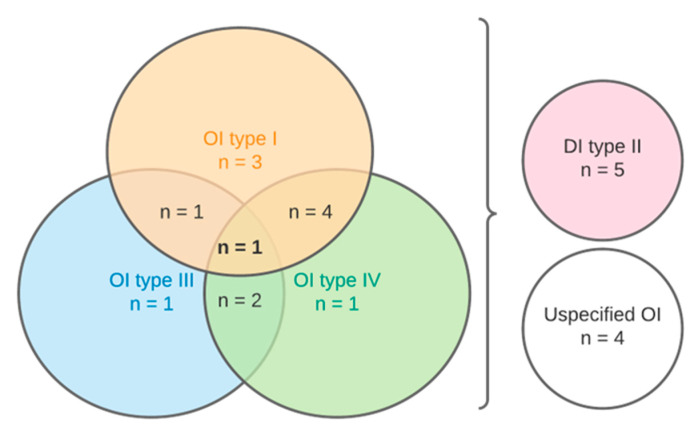
Distribution of studies based on types of osteogenesis imperfecta.

For the purpose of facilitating the result analysis, the 17 included articles (Table 3) were divided based on the type of osteogenesis imperfecta. Nine papers studied DI affected teeth in patients of type I OI, eight papers examined type IV OI and DI and five studies looked at type III OI and DI. Four papers did not classify the type of OI for samples. As there was an overlap between studies, a Venn diagram was used to clarify the duplicate number of articles, as shown in Figure 3.

Defective collagen was described using 14 parameters, which were divided into four categories: collagen characteristics (collagen organisation, diameter, shape, structure, quantity, orientation and density), collagen types (collagen types III, IV, VI and collagen pC and pN), collagen physical properties and D-banding category.

**Figure 4:**
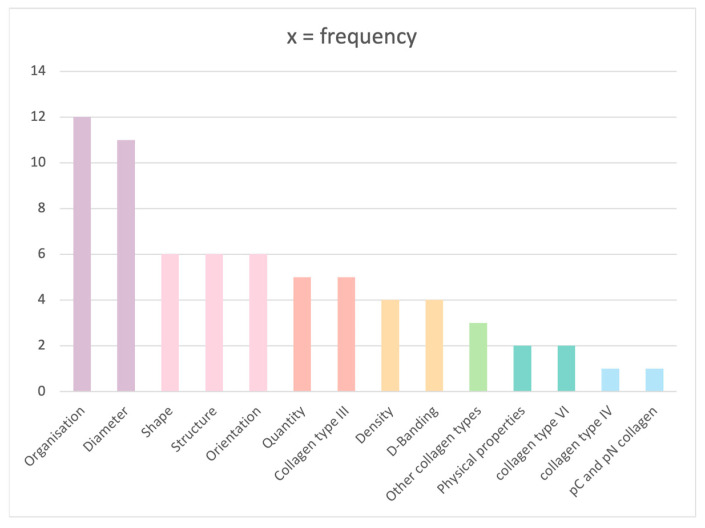
Number of studies covering each parameter.

The most frequent criteria used for assessment were fibrils organisation and collagen diameter, accounting for approximately 70% (12/17) and 65% (11/17) of the studies, respectively. Fibre shape, structure and orientation were described by 35% (6/17) of the papers, followed by 29% of the studies (5/17) covering fibre quantity and presence of collagen type III. Other less commonly used parameters were collagen density, cross-banding, physical properties such as dentin hardness and elasticity and presence of other types of collagen. Second to collagen type I, collagen type III was more commonly tested in studies than types VI and IV, accounting for n = 5 vs. 2 and 1, respectively (Figure 4).

### 3.2. Collagen Characteristics

#### 3.2.1. Collagen Organisation

**Figure 5:**
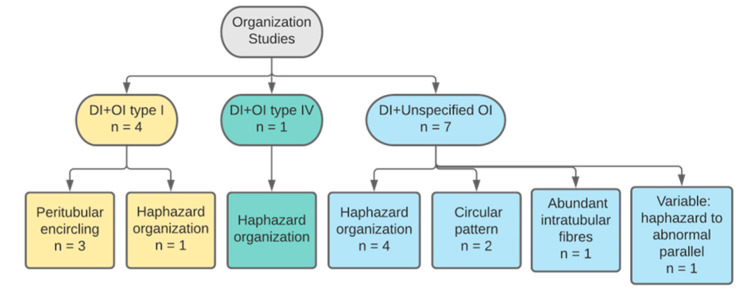
Categorisation of studies reporting on collagen organisation by DI and OI types.

**Figure 6:**
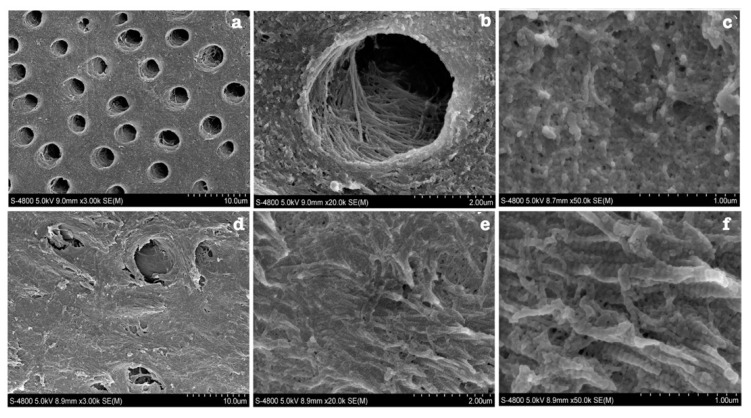
SEM image of normal dentin (**a**–**c**) and OIDI dentin **(d**–**f**). (**a**,**b**) control dentin has regular dentinal tubules and (**c**) has highly mineralised and organised collagen fibres. (**d**) Defective dentin occluded dentinal tubules (**e**,**f**) irregular organisation of exposed collagen fibres [33].

**Figure 7:**
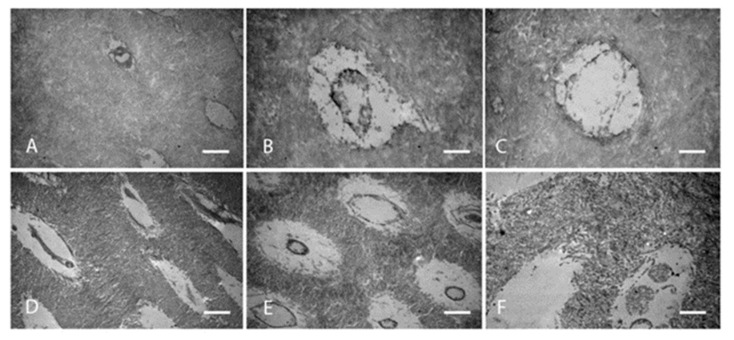
TEM images of normal and OIDI dentin. Normal condensed collagen matrix (**A**–**C**). Irregular organisation of circular collagen fibres in a loose abnormal matrix (**D**–**F**) [34].

Twelve out of 17 studies (Figure 5) reported on fibres organisation with a total of 14 primary teeth and four permanent teeth. Six studies reported on primary teeth, half of which declared the number of teeth, n = 13, primary teeth. Two studies reported on both primary and permanent teeth and only one specified the number of teeth examined, one primary and one permanent teeth. One study had a sample size of three permanent teeth. Three studies did not declare the number of teeth examined. Studies only reported on collagen of OIDI and the majority described collagen fibres as either haphazardly organised or in a form of abnormal circular bundles (Figure 6). In OI type I, fibrils were described in three studies as encircling the dentinal tubules in a transverse, cross-striated pattern [23,25,30]. An additional study reported a heterogenous organisation of the fibrils giving a completely altered and disorganised meshwork [31]. Similarly, in unspecified types of OI, DI teeth were found to have four patterns. One is altered organisation and malalignment of collagen fibres (Figure 6) [28,32,33]. Another is a characteristic circular pattern (Figure 7). These characteristic fibre bundles were found to be unorganised and unevenly distributed with large gaps and spaces [24,34]. Another study reported lack of bundle formation and instead fibres were either haphazardly arranged or forming an abnormal parallel pattern [35]. Finally, two distinctive features were also reported where fibres found lacked clear cross-striated pattern and abundant intratubular fibres [30,36]. In OI type III, one study found an occasional occurrence of parallel-aligned collagen fibres in defective atubular dentin, while in type IV OI, fibres were only described as inconsistently arranged [21,23].

#### 3.2.2. Collagen Diameter

**Figure 8:**
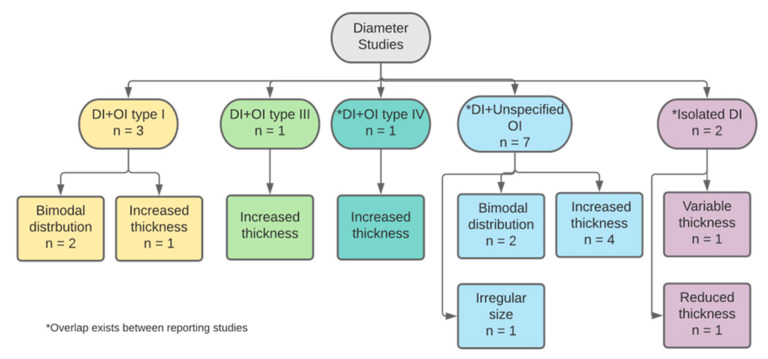
Categorisation of studies reporting on collagen diameter by DI and OI types.

The second most frequent criterion was collagen diameter. Eleven of the 17 studies (65%) described defects in collagen diameter of DI teeth as either isolated or with OI (Figure 8). Eight papers studied primary teeth and only four of which declared the number of teeth examined (n = 21 teeth). One paper examined permanent teeth and another examined both primary and permanent teeth, both of which without specifying the number of teeth examined. The eleventh study did not declare details about the type nor number of teeth.

Overall, studies described the defective dentin as having thick, coarse, abnormally enlarged collagen fibres when compared to the dentin of normal teeth. This was mostly in DI type I teeth while in isolated DI, two studies reported that fibres had reduced thickness. Normal dentin collagen fibres were found to have a diameter of 50–75 nm [31]. In OI type I, studies reported that collagen fibres did not have a uniform diameter [30,31]. In fact, they are thought to have a bimodal distribution [31]. The upper level of the abnormal thickness can reach up to 300 nm, with a median of 62.1 nm, while the lower limit median was approximately 30 nm. This was also reported by studies that examined DI teeth with unspecified OI types. The smaller fibres’ diameter ranged between 40–60 nm, while the larger population of fibres range was 80–100 nm [35,36]. In a more recent study, enlarged fibres were found to range between 81–124 nm [33]. However, most studies, from the earliest paper included to the most recently published, described defective dentin as having thick, coarse, abnormally enlarged collagen fibres that are variably increased in diameter when compared to dentin of normal teeth [23,24,28,33,34]. Similarly, in OI type III and type IV studies, defective dentin had abnormally thickened and coarse collagen fibres [25,37]. In isolated DI teeth, collagen diameter was also found to have a bimodal distribution [35]. However, the range was more divergent than in other types of DI or OI affected teeth. Thin fibrillar structures had a thickness of 10–20 nm and increased length reaching up to 1700 nm [36].

#### 3.2.3. Collagen Shape

**Figure 9:**
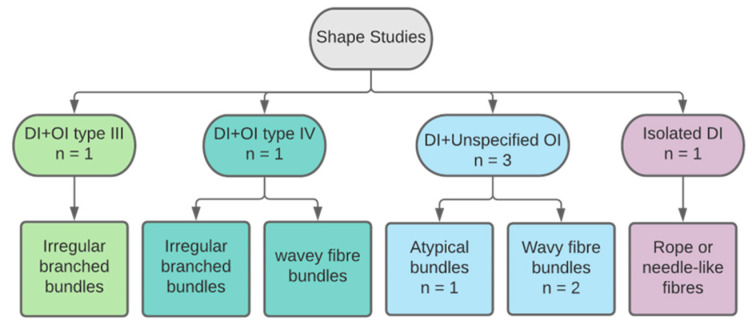
Categorisation of studies reporting on collagen shape by DI and OI types.

Altered shape of collagen fibres was described by six papers (Figure 9). Four studies reported results on primary teeth and only two declared the number of teeth, with a total sample size of 10 primary teeth. A study examined one primary tooth and an unknown number of permanent teeth. Another examined permanent teeth only. Three studies reported the following features in an unspecified type of OI: atypical recognisable collagen fibres, irregular threads and wavy formations and curved groups of cross-striated fibres [24,30,33]. In types III and IV OI, collagen fibres were found to have an extension of irregular branches [38], while in types I and IV OI, they were described as thick and wavey shapes [37]. Previous studies examined teeth with DI type I and various types of OI. One study examined teeth of isolated DI type II and found a distinctive shape of collagen fibres described as “rope or needle-like” structures [36].

#### 3.2.4. Collagen Structure

**Figure 10:**
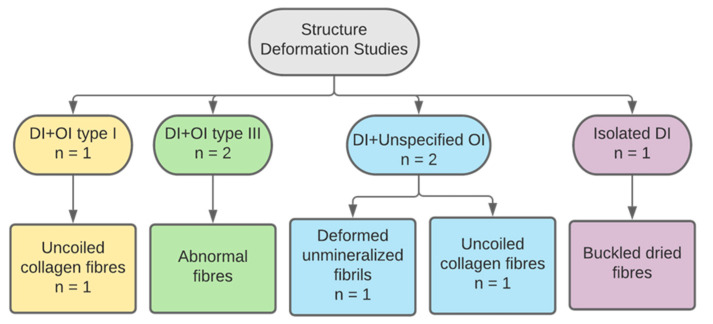
Categorization of studies reporting on collagen structural deformation by DI and OI types.

Six studies described collagen in DI teeth as structurally deformed or abnormal (Figure 10). The known sample size n = 6, three primary teeth and three permanent, was reported by three studies. Other papers did not report on sample size. A study of type I OIDI reported on the finding of unravelled helices of collagen fibres [30]. Similarly, a study of unspecified OI type reported uncoiled collagen fibres in defective dentin of OIDI teeth [35]. Another study with similar subjects found a high presence of abnormally formed collagen that was uncoated with minerals [33]. One study examined DI affected collagen in OI type III affected subjects and reported the presence of altered “pulsed” formations of abnormal collagen fibrils [25].

Another study that also examined OI type III affected subjects found abnormal quality and structure of collagen fibres [27]. The last paper studied isolated DI teeth and reported abnormal collagen structure that led to buckling of the fibres when dried [32].

#### 3.2.5. Collagen Quantity

Five studies examined the quantity of collagen fibres in OIDI affected teeth, none of which specified a description for DI collagen defects in type I OI. All studies reported results on primary teeth, three of which declared the number of teeth, with a total sample size of five primary teeth. One paper also studied an additional permanent tooth [21]. Three papers did not specify the exact type of OI affecting the subjects [24,25,28], while two studies specified their results for OI types III and IV [21,27]. Despite the variation of the type of disorder affecting the subjects, all samples of DI defective dentin showed similar findings. Collagen fibre quantity was reduced in all study groups when compared to controls [21,24,25,27,28].

#### 3.2.6. Collagen Orientation

**Figure 11:**
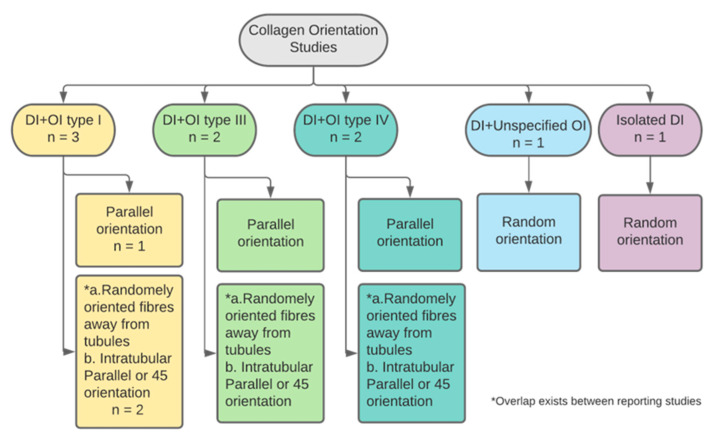
Categorisation of studies reporting on collagen orientation by DI and OI types.

Six studies reported on collagen fibres orientation in DI affected teeth (Figure 11). Four papers reported on primary teeth yet only three specified the number of teeth, n = 18 [25,26,28,37]. One study reported on both dentitions without sample size specifications and another did not declare any information on study sample. One paper examined DI teeth in subjects affected by types I, III and IV OI, all of which had comparable fibres orientation. The direction of orientation differed according to proximity to the dentinal tubules. Intratubular fibres were found mostly parallel to the long axis of the tubule, while in sections away from dentinal tubules fibres were in random orientations [26]. Occasional parallel orientation was also found in DI teeth affected by OI type III [23]. This was also reported by a study that examined type I OIDI affected teeth, with the addition of 45° orientation of some intratubular fibres [25]. One study reported a unidirectional parallel fibre orientation in types I and IV OI-DI teeth [37]. In contrast, a more recent study found that the orientation of collagen fibres is mostly disoriented and haphazard [28]. This was also the finding of a study that examined teeth of isolated DI type II; collagen fibres did not follow a specific orientation and were mostly aberrant [35].

#### 3.2.7. Collagen Density

Collagen fibres density was described by four studies. The known sample size was n = 3 primary teeth, reported by two studies. Other papers did not report on sample size. In general, three papers examined primary teeth and one additionally examined permanent teeth. The fourth study examined permanent teeth only. In type I OI, a study reported increased density in collagen bundles to a degree that single fibres forming the bundle were undistinguishable [30]. However, this finding was in contrary with other studies’ results. Reduced fibre density and loose collagen bundles were common findings in multiple studies, one of which examined OI types III and IV affected DI teeth [33,34,38].

### 3.3. Collagen Types

#### 3.3.1. Collagen Type III

**Figure 12:**
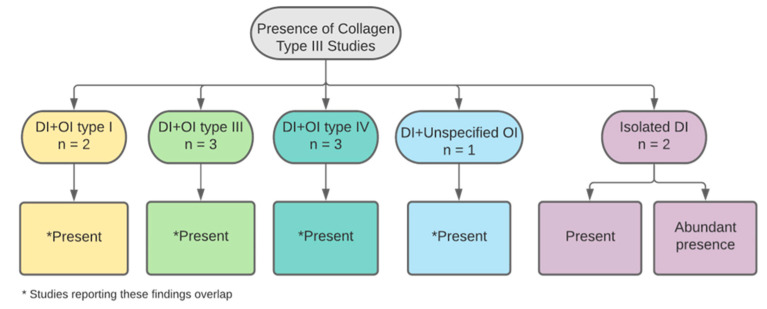
Categorisation of studies reporting on collagen type III by DI and OI types.

The presence of type III collagen fibres was tested by five studies (Figure 12). Four studies reported on primary teeth, with a total sample size eight primary teeth declared by two papers. Two papers additionally examined permanent teeth without declaring the number of teeth and one study did not declare the dentition examined, nor was the sample size and high staining for these fibres evident in all DI type I affected teeth, including OI types I, III and IV [23,26,36,38]. One of these studies found an association between evidence of type III collagen and poor mineralisation [26]. Another paper found an increased availability for type III collagen fibres in isolated DI than OIDI [36]. A description of the fibres was provided as having a “fan-like” layered structure that was more frequently dense in the periphery of the defective dentin. This was found in DI teeth of subjects affected by type III and IV OI [38].

#### 3.3.2. Collagen Types IV and VI

The presence of other types of collagen such as types IV and VI was documented by three papers. The sample size documented by these papers is unknown. In general, all examined primary teeth and one additionally examined permanent teeth. In types I and IV OIDI teeth, intense staining for collagen type VI was evident yet more significant in type IV OI subjects [36]. This finding was also supported by another study where results state that collagen type VI was detected in all OIDI affected teeth [24]. In isolated DI on the other hand, type IV collagen was found as thin fibrillar structures in the dentin matrix [35].

#### 3.3.3. pC-Collagen and pN-Collagen

One paper found that dentin of isolated DI affected teeth stained heavily for C-terminal peptide region of type I fibres but much less for N-terminal peptide region. The study was conducted on primary teeth of an unknown number. Their interpretation was that the heavy staining for C-terminal peptide and the lack of staining for the N-terminal peptide mean proper formation of mature collagen fibres, since the latter region is mostly cleaved away during collagen formation [36]. Therefore, the reduction in fibre diameter in isolated DI reported by other studies is not a result of immature fibre formation.

#### 3.3.4. Other Types of Collagen

Two other studies reported unusual types of collagen found in OIDI affected teeth. Both studies examined primary teeth; one declared n = 2 primary teeth. A study of Type I OI, DI teeth found excessively large diameter fibres or hyperfibres that reach about 300 nm in thickness [37]. These extraordinarily coarse hyperfibres were also found in another study of OIDI teeth with unspecified OI type [35]. Other forms of collagen as symmetrical collagen segments (SCSs) and fibrous long-spacing collagen (FLS) were found in OIDI teeth of unspecified OI type. Their dimensions are at least double in size than native collagen fibres, at about 265 nm length × 950 nm thickness. Although they have similar dimensions, their periodicity varied. SCSs have a periodicity of approximately 55 nm, while FLS is about 125 nm. This study however specified that type IV OI-DI teeth exhibited more FLS than other types [39].

### 3.4. Physical Properties

Two studies only examined physical properties of defective dentin in DI teeth and both papers tested dentin hardness and elasticity. The total sample size n = 9 primary teeth. One study reported, in type III OI, DI teeth exhibited significantly lower dentin hardness and elasticity than normal dentin [27]. The other study examined type I OI, DI teeth and reported 50% reduction in dentin hardness when compared to control teeth. This study also found that modulus of elasticity had a bimodal distribution, because defective collagen fibres were divided into large and small diameters. The higher-end fibres had an elastic modulus of approximately 4 GPa, while the fibres in the lower end had an elastic modulus of approximately 10 GPa [31].

### 3.5. D-Banding Periodicity

**Figure 13:**
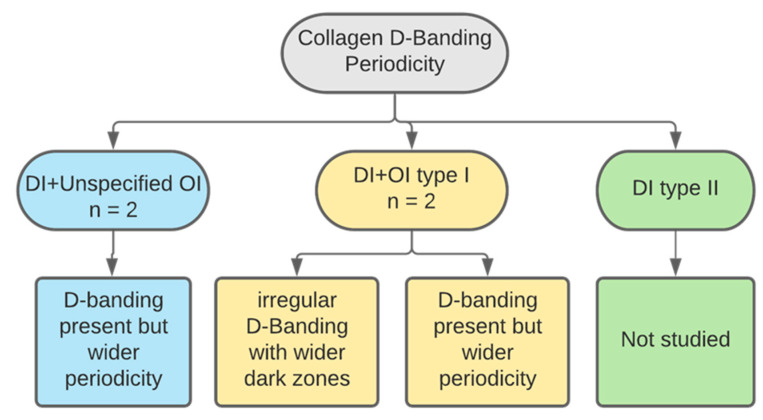
Categorisation of studies reporting on collagen D-banding by DI and OI types.

**Figure 14:**
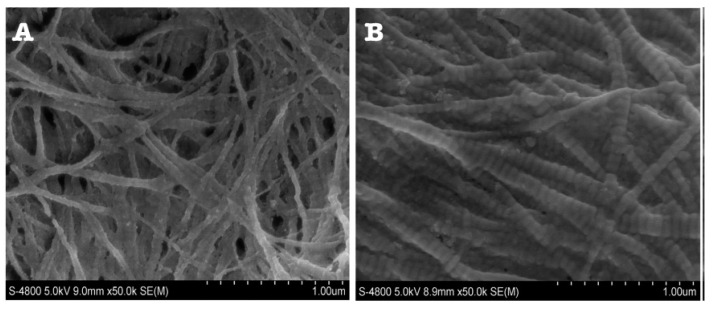
SEM of collagen fibres. Normal and intact fibres with normal banding periodicity in control dentin (**A**). DI dentin collagen fibres have variable and wide banding periodicity (**B**) [33].

A total of four studies described the ultrastructural collagen characteristic D-banding (Figure 13). Three papers examined primary teeth, n = 18 primary teeth [31,37,39]. One examined permanent teeth without specification of sample size. Two studied type I OI affected teeth, one of which reported the presence of a few sporadic D-banding periodicity. However, the majority of the fibres exhibited normal D-banding periodicity but of abnormal distance. It was found that the spacing was larger than in normal teeth, measuring between 50–80 nm versus 52–75 in healthy dentin [31]. The other study reporting on type I OI found that although the periodicity of the fibres was as the control teeth, the D-banding could not be seen. Instead, only larger dark areas and narrower light areas were observed [37]. The findings of the first study were also reported by the remaining two papers. In unspecified OI types, wider periodicity of defective collagen was found, ranging between 58.4–70.2 versus 55.9–67.9 in normal collagen (Figure 14) [33,39].

### 3.6. Isolated DI versus OIDI

Five studies reported on isolated dentinogenesis imperfecta; among those, five criteria can be described and compared with syndromic DI or OIDI. The criteria were collagen diameter, shape, orientation, presence of collagen type III and mineralisation. In isolated DI, fibres had a range of variable diameter, which expands increasingly and decreasingly from the normal range, but fibres were generally described as large and coarse [33,34,35]. This was also reported in OIDI teeth. However, in isolated DI, an additional description was found on the presence of thin fibrillar structures [35,36]. With regards to shape observations, in isolated DI, thin needle-like fibrils were found, which was not seen in the dentin of OIDI teeth [35]. The presence of these needle-like structures can be correlated with the decrease in fibril diameter and presence of thin fibrillar structures. The orientation of collagen fibres in isolated DI teeth was described as abnormal, with no further clarification on whether the direction was different intra- and intertubular, as in OIDI teeth [35]. In the latter, studies found that parallel fibrils were occasionally found in the intratubular dentin [25,26]. The presence of collagen type III was tested in both isolated DI teeth and OIDI teeth. Reactivity to this type of collagen was found in all teeth specimens [26,35,38]. However, one study reported higher reactivity in isolated DI teeth when compared to OIDI teeth and control teeth [35,36]. Finally, in both types of DI, studies reported reduced mineralisation of collagen fibrils. More specifically, in isolated DI, intrafibrillar mineralisation was absent [32,33].

## 4. Discussion

DI is a genetic disorder that renders affected teeth with aesthetic and functional defects. The disease presents with a range of features, including greyish-brown discolouration, pulpal obliteration, crown fractures and severe attrition [15]. Clinically, it can affect both primary and permanent dentitions. Management of DI is thought to be challenging. The American Academy for Paediatric Dentistry (AAPD) recommends early management of children affected by DI with preventive measures. Aspects of management include tooth structure preservation and aesthetic improvement [40]. Studies recommend placement of stainless-steel crowns over primary molars and composite restoration build-ups for anterior teeth incisors and canines [41]. However, the success of composite restorations depends on the hybrid layer formed by the interdigitation of the composite restoration, adhesive resin and collagen fibres. Due to the presence of abnormal collagen fibres in the dentin of DI teeth, the adhesive system is not able to infiltrate the collagen layer exposed after demineralisation. This leads to incomplete immersion of collagen fibres in the adhesive resin system [3]. The hybrid layer formed is of questionable quality as the exposed collagen fibres are prone to hydrolysis by endogenous enzymes, leading to degradation of the hybrid layer and subsequent failure of adhesive restoration [34]. The use of adhesive systems that have the capability to form chemical bonds with collagen fibres is an area of research for the future of restorative dentistry in DI patients. For this reason, understanding the microscopic changes in collagen is of clinical importance.

Studies of dental defects due to DI are numerous. However, the literature is limited on the defects of collagen in DI teeth. This systematic review identified 14 abnormal changes to clarify the deformity in collagen of DI teeth and examine its ultrastructural defects. Increased collagen diameter and disorganised fibres were the most common defects found in studies. A qualitative examination of collagen fibres showed that, although smaller sized fibres were occasionally found, thick coarse collagen fibres were a common finding in defective dentin of DI teeth with both isolated DI and OIDI. The organisation of collagen fibres in both DI types was found to be irregular, haphazard and with an abnormal circular pattern. Other commonly described changes in isolated DI and OIDI were reduced collagen quantity, abnormal fibres’ shape and having a parallel to random orientation [24,28].

Among the 12 studies that described changes in collagen diameter, five studies explained the presence of both smaller sized fibres and thick coarse collagen fibres [30,31,35,36]. Two of these studies also examined the presence of collagen type III fibres [35,36]. Collagen type III is similar to type I, as both are fibril forming collagens rather than hexagonal or network forming types. However, type III is much less abundant than type I and is rarely found in normal dentin. All studies that investigated collagen type III found high presence of these fibres in all DI types. However, higher abundance was found to be related to DI type II teeth. The presence of collagen type III and its role in disease pathophysiology is not entirely known but it is thought that these fibres may cause abnormal mineralization of the dentin matrix. Furthermore, correlation was found between the presence of type III fibres and the small fibrillar structures. Another study suggested that these fine fibrils could be immature collagen type I fibres [31]. In dentin of normal teeth, collagen type III fibres are covered by those of type I. Therefore, the presence of type III collagen fibres could be due to defective formation of type I fibres exposing those of type III to detective stains and antibodies. Consequently, type III collagen is not necessarily present due to a defect in dentin formation, as it may be present in normal dentin yet undetectable [36].

The disorganisation of collagen fibres was described by twelve studies [21,23,24,25,28,30,31,32,33,34,35]. Fibres were described as irregularly organised, assembling in abnormal circular patterns and in lax derangements with large gaps [25,32,34]. In normal dentin, collagen acts as a scaffold for mineral deposition and crystal growth. Accordingly, one study suggested a negative effect on crystals growth, given that these large spaces offset the geometrical limitation set by the scaffold allowed overgrowth of hydroxyapatite crystals [32].

In normal dentin, collagen fibres are mostly found and arranged in intertubular dentin. Peritubular dentin, on the other hand, is mainly formed of non-collagenous matrix proteins and naturally inside the tubules lays the odontoblastic process [42]. In OIDI dentin, as fibres organisation varies, lateral intratubular fibres were abundant [30,37]. This can also explain how parts of the defective dentin lack odontoblastic processes and the dentinal tubules are overruled by collagen fibres [34].

Other deformities, such as low collagen density, reduced fibres quantity and irregular shapes of collagen fibres, can be considered factors in the reduced dentin hardness found and reduced modulus of elasticity [27,31]. The reduced elasticity of peritubular collagen fibres is thought to be the first evidence in understanding the structural defects in OIDI teeth [31]. In isolated DI, studies did not report on the mechanical strength of dentin nor collagen, although it can be inferred from studies on OIDI teeth that both are reduced for the same reasons. However, one study correlated the reduced intratubular mineralisation with decreased resistance to bending stresses, which are observed with excessive drying [32].

Ultrastructural defects were reported by seven studies [27,30,31,33,35,37,39], three of which described structural defects while the remaining examined D-banding periodicity [31,33,37,39]. The deformities found were loss of triple helix structure and uncoiled collagen fibres [35,36]. The fibre uncoiling is normally seen at the end of the collagen fibres [43]. In this case, the uncoiling was seen throughout the entire length of the fibre, suggesting abnormal structure due to defective fibrillogenesis [30].

Increased D-banding periodicity was a common finding in four different studies. D-banding periodicity is equal to 67 nm in normal dentin. It exists because the length of tropocollagens is about four times longer than their periodicity, meaning that L = 4.46D, giving areas of gaps (0.54D) and overlaps (0.46D), and when combined they account for the 67 nm D-periodicity. In normal dentin, these gaps and overlaps are nucleating sites for mineral deposition and crystal growth [44]. However, fewer mineral depositions were found in DI teeth when compared to normal teeth, suggesting that normal D-banding periodicity is a prerequisite for mineral deposition [33].

Abnormal characteristic collagen types have been reported by multiple studies. These include SCS, FLS and hyperfibres [35,37,39]. The significantly thick hyperfibres were found away from the dentinal tubules in the loose dentinal matrix of OIDI teeth [35,37], while FLS and SCS collagen forms were found inside the dentinal tubules. Both forms were evident in teeth of OIDI with an unspecified type of OI. However, the FLS collagen was specifically related to type IV OI. It was also reported by the study that the SCS collagen is unrelated to the formation of defective dentin matrix and that SCS does exist in normal dentin. FLS relation to OIDI on the other hand is still unconfirmed [39].

## 5. Conclusions

This systematic review reports changes in collagen of teeth affected by dentinogenesis imperfecta. The most frequently found macro-deformities were coarse collagen fibres, decreased fibres quantity, random to parallel fibres orientation and irregular organisation. Ultrastructural defects were uncoiled collagen fibres and increased D-banding periodicity. In addition to the presence of types III, IV and VI collagen fibres, hyperfibres, SCS and FLS collagen forms were found. Understanding the microscopic changes in collagen is of clinical importance as it will enable the innovation of adhesive systems that have the capability to form chemical bonds with collagen fibres. This is an area of research for the future of restorative dentistry in DI patients.

## Figures and Tables

**Table 1:** Updated classification of osteogenesis imperfecta.

OI Type	Gene Mutation	Severity	Clinical Presentation
Defects in collagen synthesis and structure			
I	COL1A1	Mild	- Dentinogenesis Imperfecta, uncommon but highly heritable - 50% of all OI - Blue sclera - Mild susceptibility to long bone fracture - Premature hearing loss
II	COL1A1–COL1A2	Lethal	- High mortality rate - 80% die within the first week
III	COL1A1–COL1A2	Progress with age	- Most severe but nonlethal - Skeletal deformity (scoliosis) - Blue sclera - Common presence of Dentinogenesis Imperfecta - Most cases non-ambulatory
IV	COL1A1–COL1A2	Moderately severe	- Variable presence of Dentinogenesis Imperfecta - Hearing loss - Variable skeletal malformation - Blue sclera

**Table 2:** DI classification, clinical representation and associated gene mutation [14,15,19,20].

**Shields** **1973** [15]	**Witkop** **1988** [19]	**Barron et al.** **2008** [14]	**De La Dure-Molla M et al.** **2015** [20]	**Clinical Presentation**	**Associated Gene**
Type I DI/DI associated with OI/syndromic DI	Dentinogenesis Imperfecta	DGI-I	-	- Dentition discoloration - Progressive pulpal obliteration	COL1A1 COL1A2
Type II DI	Hereditary opalescent dentin	DGI-II	Moderate isolated DI	- Dentition discoloration - Crown constriction	DSPP
Type III DI	Brandywine isolate	DGI-III	Severe isolated DI	- Shell teeth with enlarged pulps	DSPP

**Table 3:** Included studies, date of publication, type of OI and collagen parameter studies.

Author(s)	Date	Study Design	Dentition	DI Type	OI Type	Parameter Studied
Intarak et al. [21]	2020	Cross sectional	1 primary and 1 permanent teeth	Type I	Type IV	Collagen quantity Collagen organization
Ranta et al. [23]	1993	Review	Unspecified	Types I and II	Types I and III	Collagen orientation Collagen diameter Collagen organization Type III presence
Orsini et al. [24]	2014	Cross sectional	Primaryunspecified number	Type I	Type I	Collagen quantity Collagen diameter Collagen organization Presence of type VI
Hall et al. [25]	2002	Cross sectional	Primary and permanentunspecified number	Types I and II	Types I, III and IV	Collagen Quantity Collagen diameter Collagen orientation Collagen organization Structural deformation
Majorana et al. [26]	2010	Cross sectional	7 Primary teeth	Type I	Types I, III and IV	Collagen orientation Presence of type III collagen
Budsamongkol et al. [27]	2019	Cross sectional	1 primary tooth	Type I	Type III	Collagen quantity Structural deformation Dentin hardness
Nutchoey et al. [28]	2021	Cross sectional	3 primary teeth	Type I	unspecified	Collagen quantity Collagen diameter Collagen organization Collagen orientation
Waltimo et al. [30]	1996	Cross sectional	Primary and permanentunspecified number	Type I	Type I	Collagen diameter Collagen shape Collagen organization Collagen density Structural deformation
Ibrahim et al. [31]	2019	Cross sectional	8 Primary teeth	Type I	Type I	Collagen D-banding Collagen organization Collagen diameter Physical properties
Kinney et al. [32]	2001	Cross sectional	3 permanent teeth	Type II	unclear	Structural deformation Collagen organization
Duan et al. [33]	2016	Cross sectional	28 Permanent teeth	Type I	unspecified	Collagen diameter Collagen shape Collagen organization Collagen density D-banding Structural deformation
Josic et al. [34]	2020	Cross sectional	Primaryunspecified number	Type I	Unspecified	Collagen diameter Collagen organization Collagen density
Waltimo et al. [35]	1995	Cross sectional	Primaryunspecified number	Types I and II	Types I and IV	Collagen diameter Collagen shape Collagen organization Presence of types III and VI and pC-collagen and pN-collagen
Waltimo et al. [36]	1994	Review	Primary and permanentunspecified number	Types I and II	Types I and IV	Collagen diameter Collagen orientation Collagen organization Types III and IV presence Structural deformation Hyperfibres
Waltimo [37]	1994	Case series	8 primary teeth	Type I	Types I and IV	Collagen diameter Collagen shape Collagen orientation Hyperfibres D-banding
De Coster et al. [38]	2006	Cross sectional	Primary and permanentunspecified number	Type I	Types III and IV	Collagen shape Collagen density Type III presence and density
Waltimo [39]	1996	Cross sectional	2 primary teeth	Type I	Types I and IV	Collagen D-banding SCS (symmetrical collagen segments) FLS (fibrous long-spacing collagen)

## Data Availability

Not applicable.

## Supplementary Materials

The following supporting information can be downloaded at: https://www.mdpi.com/article/10.3390/dj11040095/s1, Supplementary Table S1 (Categories of search terms and their data base); Supplementary Data S1 (Data Collection Form) and Supplementary Data S2 (Systematic Review Search Strategy).

## References

[1] Gadi L. (2022). Morphological and Ultrstructural Collagen Defects: Impact and Implications in Dentinogenesis Imperfecta. Ph.D. Thesis.

[2] Boskey A.L. (2013). Bone composition: Relationship to bone fragility and antiosteoporotic drug effects. Bonekey Rep..

[3] Breschi L., Maravic T., Cunha S.R., Comba A., Cadenaro M., Tjäderhane L., Pashley D.H., Tay F.R., Mazzoni A. (2018). Dentin bonding systems: From dentin collagen structure to bond preservation and clinical applications. Dent. Mater..

[4] Myllyharju J., Kivirikko K.I. (2001). Collagens and collagen-related diseases. Ann. Med..

[5] Gelse K., Pöschl E., Aigner T. (2003). Collagens—Structure, function, and biosynthesis. Adv. Drug Deliv. Rev..

[6] Rauch F., Glorieux F.H. (2004). Osteogenesis imperfecta. Lancet.

[7] Martin E., Shapiro J.R. (2007). Osteogenesis imperfecta: Epidemiology and pathophysiology. Curr. Osteoporos. Rep..

[8] Lindahl K., Åström E., Rubin C.-J., Grigelioniene G., Malmgren B., Ljunggren Ö., Kindmark A. (2015). Genetic epidemiology, prevalence, and genotype–phenotype correlations in the Swedish population with osteogenesis imperfecta. Eur. J. Hum. Genet..

[9] Sillence D.O., Senn A., Danks D.M. (1979). Genetic heterogeneity in osteogenesis imperfecta. J. Med. Genet..

[10] Marini J.C., Cabral W.A., Thakker R.V., Whyte M.P., Eisman J.A., Igarashi T. (2018). Chapter 23—Osteogenesis Imperfecta. Genetics of Bone Biology and Skeletal Disease.

[11] Forlino A., Cabral W.A., Barnes A.M., Marini J.C. (2011). New perspectives on osteogenesis imperfecta. Nat. Rev. Endocrinol..

[12] Opsahl Vital S., Gaucher C., Bardet C., Rowe P.S., George A., Linglart A., Chaussain C. (2012). Tooth dentin defects reflect genetic disorders affecting bone mineralization. Bone.

[13] Pallos D., Hart P.S., Cortelli J.R., Vian S., Wright J., Korkko J., Brunoni D., Hart T.C. (2001). Novel COL1A1 mutation (G599C) associated with mild osteogenesis imperfecta and dentinogenesis imperfecta. Arch. Oral Biol..

[14] Barron M.J., Mcdonnell S.T., Mackie I., Dixon M.J. (2008). Hereditary dentine disorders: Dentinogenesis imperfecta and dentine dysplasia. Orphanet J. Rare Dis..

[15] Shields E., Bixler D., El-Kafrawy A. (1973). A proposed classification for heritable human dentine defects with a description of a new entity. Arch. Oral Biol..

[16] Witkop C.J. (1975). Hereditary defects of dentin. Dent. Clin. N. Am..

[17] Gupta S.K., Saxena P., Jain S., Jain D. (2011). Prevalence and distribution of selected developmental dental anomalies in an Indian population. J. Oral Sci..

[18] Cassia A., Aoun G., El-Outa A., Pasquet G., Cavézian R. (2017). Prevalence of Dentinogenesis imperfecta in a French population. J. Int. Soc. Prev. Community Dent..

[19] Witkop C.J. (1988). Amelogenesis imperfecta, dentinogenesis imperfecta and dentin dysplasia revisited: Problems in classification. J. Oral Pathol. Med..

[20] De La Dure-Molla M., Philippe Fournier B., Berdal A. (2015). Isolated dentinogenesis imperfecta and dentin dysplasia: Revision of the classification. Eur. J. Hum. Genet..

[21] Intarak N., Budsamongkol T., Theerapanon T., Chanamuangkon T., Srijunbarl A., Boonprakong L., Porntaveetus T., Shotelersuk V. (2020). Tooth ultrastructure of a novel *COL1A2* mutation expanding its genotypic and phenotypic spectra. Oral Dis..

[22] Macdougall M., Dong J., Acevedo A.C. (2006). Molecular basis of human dentin diseases. Am. J. Med. Genet. Part A.

[23] Ranta H., Lukinmaa P.L., Waltimo J. (1993). Heritable dentin defects: Nosology, pathology, and treatment. Am. J. Med. Genet..

[24] Orsini G., Majorana A., Mazzoni A., Putignano A., Falconi M., Polimeni A., Breschi L. (2014). Immunocytochemical detection of dentin matrix proteins in primary teeth from patients with dentinogenesis imperfecta associated with osteogenesis imperfecta. Eur. J. Histochem..

[25] Hall R.K., Manière M.C., Palamara J., Hemmerlé J. (2002). Odontoblast dysfunction in osteogenesis imperfecta: An LM, SEM, and ultrastructural study. Connect. Tissue Res..

[26] Majorana A., Bardellini E., Brunelli P.C., Lacaita M., Cazzolla A.P., Favia G. (2010). Dentinogenesis imperfecta in children with osteogenesis imperfecta: A clinical and ultrastructural study. Int. J. Paediatr. Dent..

[27] Budsamongkol T., Intarak N., Theerapanon T., Yodsanga S., Porntaveetus T., Shotelersuk V. (2019). A novel mutation in COL1A2 leads to osteogenesis imperfecta/Ehlers-Danlos overlap syndrome with brachydactyly. Genes Dis..

[28] Nutchoey O., Intarak N., Theerapanon T., Thaweesapphithak S., Boonprakong L., Srijunbarl A., Porntaveetus T., Shotelersuk V. (2021). Phenotypic features of dentinogenesis imperfecta associated with osteogenesis imperfecta and *COL1A2* mutations. Oral Surg. Oral Med. Oral Pathol. Oral Radiol..

[29] Cochrane Effective Practice and Organisation of Care (EPOC) Data Collection Form. EPOC Resources for Review Authors, 2013. epoc.cochrane.org/resources/epoc-specific-resources-review-authors.

[30] Waltimo J., Ojanotko-Harri A., Lukinmaa P.L. (1996). Mild forms of dentinogenesis imperfecta in association with osteogenesis imperfecta as characterized by light and transmission electron microscopy. J. Oral Pathol. Med..

[31] Ibrahim S., Strange A.P., Aguayo S., Shinawi A., Harith N., Mohamed-Ibrahim N., Siddiqui S., Parekh S., Bozec L. (2019). Phenotypic Properties of Collagen in Dentinogenesis Imperfecta Associated with Osteogenesis Imperfecta. Int. J. Nanomed..

[32] Kinney J.H., Pople J.A., Driessen C.H., Breunig T.M., Marshall G.W., Marshall S.J. (2001). Intrafibrillar mineral may be absent in dentinogenesis imperfecta type II (DI-II). J. Dent. Res..

[33] Duan X., Liu Z., Gan Y., Xia D., Li Q., Li Y., Yang J., Gao S., Dong M. (2016). Mutations in *COL1A1* gene change dentin nanostructure. Anat. Rec..

[34] Josic U., Maravic T., Bossù M., Cadenaro M., Comba A., Ierardo G., Polimeni A., Florenzano F., Breschi L., Mazzoni A. (2020). Morphological Characterization of Deciduous Enamel and Dentin in Patients Affected by Osteogenesis Imperfecta. Appl. Sci..

[35] Waltimo J., Ranta H., Lukinmaa P.L. (1995). Ultrastructure of dentin matrix in heritable dentin defects. Scanning Microsc..

[36] Waltimo J., Risteli L., Risteli J., Lukinmaa P.L. (1994). Altered collagen expression in human dentin: Increased reactivity of type III and presence of type VI in dentinogenesis imperfecta, as revealed by immunoelectron microscopy. J. Histochem. Cytochem..

[37] Waltimo J. (1994). Hyperfibers and vesicles in dentin matrix in dentinogenesis imperfecta (DI) associated with osteogenesis imperfecta (OI). J. Oral Pathol. Med..

[38] De Coster P.J., Cornelissen M., De Paepe A., Martens L.C., Vral A. (2007). Abnormal dentin structure in two novel gene mutations [COL1A1, Arg134Cys] and [ADAMTS2, Trp795-to-ter] causing rare type I collagen disorders. Arch. Oral Biol..

[39] Waltimo J. (1996). Unusual forms of collagen in human dentin. Matrix Biol..

[40] Council O. (2013). Guideline on dental management of heritable dental developmental anomalies. Pediatr. Dent..

[41] Frassetto A., Breschi L., Turco G., Marchesi G., Di Lenarda R., Tay F.R., Pashley D.H., Cadenaro M. (2016). Mechanisms of degradation of the hybrid layer in adhesive dentistry and therapeutic agents to improve bond durability—A literature review. Dent. Mater..

[42] Goldberg M., Kulkarni A.B., Young M., Boskey A. (2011). Dentin: Structure, composition and mineralization. Front. Biosci. Elite Ed..

[43] Holbrook K.A., Byers P.H. (1987). Diseases of the extracellular matrix: Structural alterations of collagen fibrils in skin. Connective Tissue Disease Molecular Pathology of the Extracellular Matrix.

[44] Nudelman F., Pieterse K., George A., Bomans P.H., Friedrich H., Brylka L.J., Hilbers P.A., De With G., Sommerdijk N.A. (2010). The role of collagen in bone apatite formation in the presence of hydroxyapatite nucleation inhibitors. Nat. Mater..

