# Supplementary material for: Morphological and Ultrastructural Collagen Defects: Impact and Implications in Dentinogenesis Imperfecta
Source: Dent J (Basel). 2023 Apr 3;11(4):95. doi: 10.3390/dj11040095 (PMC10137525; doi:10.3390/dj11040095)
Supplement: Supplementary file 1 [file dentistry-11-00095-s001.zip › dentistry-2055921-supplementary.pdf]

Supplementary Table S1. Categories of search terms and their data base.

	Database	DI	OI	Collagen
PubMed	Controlled vocabulary (MeSH terms)	Capdepont Teeth<br>Dentinogenesis Imperfecta<br>Opalescent Dentin	Brittle Bone<br>Fragilitas Ossium<br>Lobstein* Disease<br>Osteogenesis Imperfecta	Collagen
PubMed	Free text terms	Capdepont teeth<br>Capdepont tooth<br>Dentinogenesis Imperfecta<br>Dentin* Dysplasia<br>Dentin* Sialophosphoprotein<br>Opalescent Dentin*<br>Opalescent teeth/tooth	Brittle bone<br>Bruck syndrome*<br>Fragilitas Ossium<br>Fibrogenesis imperfecta ossium<br>Lobstein* Disease<br>Osteogenesis Imperfecta<br>Osteopsathyrosis*	Collagen<br>Collagen fib*<br>COL1A1<br>COL1A2<br>Collagen type Ⅰ<br>Collagen type Ⅱ<br>Collagen type Ⅳ
OVID	Controlled vocabulary (MeSH terms)	Dentin* Dysplasia<br>Dentinogenesis Imperfecta	Osteogenesis Imperfecta	Collagen<br>Collagen type Ⅰ<br>Collagen type Ⅱ<br>Collagen type Ⅳ
OVID	Free text terms	Dentinogenesis Imperfecta<br>Dentin* Dysplasia<br>Dentin* Sialophosphoprotein	Brittle bone<br>Bruck syndrome*<br>Fragilitas Ossium<br>Fibrogenesis imperfecta ossium	Collagen<br>Collagen fib*<br>COL1A1<br>COL1A2

		Opalescent Dentin*<br>Opalescent teeth<br>Opalescent tooth	Lobstein* Disease<br>Lobstein* syndrome<br>Osteogenesis Imperfecta<br>Osteopsathyrosis*	Collagen type Ⅰ<br>Collagen type Ⅱ<br>Collagen type Ⅳ
Teeth	MeSH teeth abnormality	Teeth tooth dentin*		
Subdo-main	bond, mutant, disorder, genotype			

Supplementary Data S1. Data Collection Form

THE COCHRANE COLLABORATION®

Data collection form

Review title or ID

Study ID *(surname of first author and year first full report of study was published e.g. Smith 2001)*

1. General Information

1. **Date form completed** *(dd/mm/yyyy)*	
2. **Name/ID of person extracting data**	Lubabah Gadi
3. **Report author contact details**	
4. **Publication type** *(e.g. full report, abstract, letter)*	Full report
5. **Study funding source** *(including role of funders)*	
**Possible conflicts of interest** *(for study authors)*	declared as non present
6. **Notes:**	

2. Reference Details:

1. **Authors**		
2. **Year of Publication**		
3. **Journal details**	**Name**	
	**Volume**	
	**Issue**	
	**Pages**	

3. Eligibility

Study Characteristics	Review Inclusion Criteria	Location in text
7. **Type of study**	rev	--
	Case report	
	Cross sectional /Laboratory	
8. **Dental collagen tested:**	Not specified	
9. **Types of lab test**	•	
10. **Types of statistical test**	None	
11. **Types of outcome measures**	•	
12. **Decision:**	Included.	
13. **Reason for exclusion**	--	
14. **Notes:**	collagen ultrastructure not studied. Implication on collagen defect only	

DO NOT PROCEED IF STUDY EXCLUDED FROM REVIEW

4. Methods

	Descriptions as stated in report/paper	Location in text
15. **Aim of study**		
16. **Demineralization protocol**		
17. **Examination tool**		
18. **Control teeth present?**		
19. **Notes:**		

5. Participants Population and setting

	Description	Location in text
20. **Population description**		Pg.
21. **Inclusion criteria**	collagen study of teeth with DI	Pg.
22. **Exclusion criteria**	-	
23. **Method/s of recruitment of participants**	Undeclared (assume convenient sample?)	
24. **Age**		Pg.
25. **Sex**		Pg
26. **DI type?**		Pg.
27. **OI presence (if yes, type?)**		Pg.
28. **Co-morbidities**		Pg.
29. **Primary or permanent teeth:**		Pg.
30. **Control teeth**		
31. **Type of collagen examined**		Pg.
32. **Notes:**		

6. Results

	Description as stated in report/paper		Location in text
33. **Outcome name**			Pg.
34. **Outcome**			Pg.
35. **Is outcome/tool validated?**	Unclear<br>*Yes/No/Unclear*		
36. **Notes:**			

7. Applicability

37. **Does the study directly address the review question?**	No<br>*Yes/No/Unclear*	
38. **Notes:**		

Supplementary Data S2. Systematic Review Search Strategy

Date: 26/06/2020

Database: Embase <1974 to 2020 June 30>

1 ((Dentinogenesis adj3 Imperfecta) or dent* dysplasia or opalescent dentin* or opalescent teeth or opalescent tooth or dentin* sialo-phosphoprotein).mp. [mp=title, abstract, heading word, drug trade name, original title, device manufacturer, drug manufacturer, device trade name, keyword, floating subheading word, candidate term word] (1571)

2 tooth/ or dentin*/ (38074)

3 Osteogenesis Imperfecta.tw. (5600)

4 ((Osteogenesis adj3 imperfecta) or Brittle bone or Bruck syndrome* or Fibrogenesis imperfecta ossium or osteopsathyrosis or Lob-stein* disease or Lobstein* syndrome or fragilitas Ossium).mp. [mp=title, abstract, heading word, drug trade name, original title, device manu-facturer, drug manufacturer, device trade name, keyword, floating subheading word, candidate term word] (7496)

5 collagen.mp. [mp=title, abstract, heading word, drug trade name, original title, device manufacturer, drug manufacturer, device trade name, keyword, floating subheading word, candidate term word] (292263)

6 collagen fib*.mp. [mp=title, abstract, heading word, drug trade name, original title, device manufacturer, drug manufacturer, device trade name, keyword, floating subheading word, candidate term word] (30454)

7 COL1A1.mp. [mp=title, abstract, heading word, drug trade name, original title, device manufacturer, drug manufacturer, device trade name, keyword, floating subheading word, candidate term word] (5884)

8 COL1A2.mp. [mp=title, abstract, heading word, drug trade name, original title, device manufacturer, drug manufacturer, device trade name, keyword, floating subheading word, candidate term word] (2209)

9 (((((Collagen adj3 type II) or collagen) adj3 type two) or collagen) adj3 type 2).mp. [mp=title, abstract, heading word, drug trade name, original title, device manufacturer, drug manufacturer, device trade name, keyword, floating subheading word, candidate term word] (16378)

10 (((((collagen adj3 type I) or collagen) adj3 type one) or collagen) adj3 type 1).mp. [mp=title, abstract, heading word, drug trade name, original title, device manufacturer, drug manufacturer, device trade name, keyword, floating subheading word, candidate term word] (50457)

11 (((((collagen adj3 type IV) or collagen) adj3 type 4) or collagen) adj3 type four).mp. [mp=title, abstract, heading word, drug trade name, original title, device manufacturer, drug manufacturer, device trade name, keyword, floating subheading word, candidate term word] (0)

12 (Bond or Mutant or Disorder or genotype).mp. [mp=title, abstract, heading word, drug trade name, original title, device manufacturer, drug manufacturer, device trade name, keyword, floating subheading word, candidate term word] (2954792)

13 Dentinogenesis Imperfecta/ or dent* dysplasia.mp. [mp=title, abstract, heading word, drug trade name, original title, device manufacturer, drug manufacturer, device trade name, keyword, floating subheading word, candidate term word] (7386)

14 collagen type 2/ or nonfibrillar collagen/ or collagen disease/ or collagen fiber/ or collagen synthesis/ or fibrillar collagen/ or collagen defect/ or collagen type 1/ or collagen/ or collagen type 4/ or fibril associated collagen/ or collagen fibril/ (209137)

15 1 or 13 (8310)

16 3 or 4 (7496)

17 2 and 15 (825)

18 5 or 14 (292263)

19 17 and 18 (24)

20 6 or 14 (216680)

21 17 and 20 (18)

22 7 or 14 (211504)

23 17 and 22 (17)

24 8 or 14 (209942)

25 17 and 24 (18)

26 10 and 17 (8)

27 9 and 17 (0)

28 11 and 17 (0)

29 12 and 17 (221)

Annotation: ASAS the subdomain

29 = 17 (DI & teeth) + subdomains

30 18 and 29 (7)

31 7 and 29 (1)

32 22 and 29 (6)

33 8 and 29 (2)

34 24 and 29 (7)

35 9 and 29 (0)

36 10 and 29 (3)

37	11 and 29 (0)
38	6 and 29 (1)
39	15 and 16 (366)
40	2 and 39 (26)
41	18 and 40 (9)
42	6 and 40 (1)
43	7 and 40 (2)
44	22 and 40 (7)
45	8 and 40 (2)
46	24 and 40 (8)
47	9 and 40 (0)
48	10 and 40 (3)
49	11 and 40 (0)
50	7 and 17 (3)
51	8 and 17 (2)
52	6 and 17 (3)

Date: 26/06/2020

Database: Ovid MEDLINE(R) and Epub Ahead of Print, In-Process & Other Non-Indexed Citations and Daily <1946 to June 26, 2020>

1 dentin dysplasia/ or Dentinogenesis Imperfecta/ (706)

2 ((Dentinogenesis adj3 Imperfecta) or dent* dysplasia* or Hereditary Dentinogenesis Imperfecta or opalescent dentin* or opalescent teeth or opalescent tooth or dentin* sialophosphoprotein).mp. [mp=title, abstract, original title, name of substance word, subject heading word, floating sub-heading word, keyword heading word, organism supplementary concept word, protocol supplementary concept word, rare disease supplementary concept word, unique identifier, synonyms] (1852)

3 1 or 2 (1852)

4 Osteogenesis Imperfecta.tw. (4728)

5 ((Osteogenesis adj3 imperfecta) or Brittle bone or Bruck syndrome* or Fibrogenesis imperfecta ossium or Osteopsathyrosis or Lob-stein* disease or Fragilitas Ossium or Lobstein* syndrome).mp. (5897)

6 4 or 5 (5897)

7 tooth/ or dentin*/ (23597)

8 Collagen Type I/ or Collagen Type IV/ or Collagen/ or Collagen Type II/ (112224)

9 collagen.mp. [mp=title, abstract, original title, name of substance word, subject heading word, floating sub-heading word, keyword heading word, organism supplementary concept word, protocol supplementary concept word, rare disease supplementary concept word, unique identifier, synonyms] (220548)

10 collagen fib*.mp. [mp=title, abstract, original title, name of substance word, subject heading word, floating sub-heading word, key-word heading word, organism supplementary concept word, protocol supplementary concept word, rare disease supplementary concept word, unique identifier, synonyms] (21407)

11 COL1A1.mp. [mp=title, abstract, original title, name of substance word, subject heading word, floating sub-heading word, keyword heading word, organism supplementary concept word, protocol supplementary concept word, rare disease supplementary concept word, unique identifier, synonyms] (3301)

12 COL1A2.mp. [mp=title, abstract, original title, name of substance word, subject heading word, floating sub-heading word, keyword heading word, organism supplementary concept word, protocol supplementary concept word, rare disease supplementary concept word, unique identifier, synonyms] (1374)

13 (((((collagen adj3 type I) or collagen) adj3 type one) or collagen) adj3 type 1).mp. [mp=title, abstract, original title, name of substance word, subject heading word, floating sub-heading word, keyword heading word, organism supplementary concept word, protocol supplementary concept word, rare disease supplementary concept word, unique identifier, synonyms] (2412)

14 (collagen type four or collagen type 4 or collagen type IV).mp. [mp=title, abstract, original title, name of substance word, subject heading word, floating sub-heading word, keyword heading word, organism supplementary concept word, protocol supplementary concept word, rare disease supplementary concept word, unique identifier, synonyms] (5538)

15 (collagen type two or collagen type 2 or collagen type II).mp. [mp=title, abstract, original title, name of substance word, subject heading word, floating sub-heading word, keyword heading word, organism supplementary concept word, protocol supplementary concept word, rare disease supplementary concept word, unique identifier, synonyms] (6741)

16 3 and 7 (107)

17 8 or 9 (220548)

18 16 and 17 (21)

19 8 or 10 (124109)

20 16 and 19 (12)

21 8 or 11 (114017)

22 16 and 21 (12)

23 8 or 12 (112817)

24 16 and 23 (12)

25 8 or 13 (113576)

26 16 and 25 (12)

27 8 or 15 (113541)

28 16 and 27 (12)

29 8 or 14 (113253)

30 16 and 29 (12)

31 2 and 6 (283)

32 (Bond or Mutant or Disorder or genotype).mp. [mp=title, abstract, original title, name of substance word, subject heading word, floating sub-heading word, keyword heading word, organism supplementary concept word, protocol supplementary concept word, rare disease supplementary concept word, unique identifier, synonyms] (1457933)

33 16 and 32 (15)

34	17 and 33 (3)
35	19 and 33 (3)
36	21 and 33 (3)
37	23 and 33 (3)
38	25 and 33 (3)
39	27 and 33 (3)
40	29 and 33 (3)
41	3 and 6 and 7 (13)
42	17 and 41 (4)
43	19 and 41 (3)
44	21 and 41 (3)
45	23 and 41 (3)
46	25 and 41 (3)
47	27 and 41 (3)
48	29 and 41 (3)

Date: Done on 26/06/2020 and re-run on 30/06/2020.

Database: PubMed Medline

1 (((((((Dentinogenesis Imperfecta[Text Word]) OR Opalescent Dentin*[Text Word]) OR Opalescent tooth[Text Word]) OR Opalescent teeth[Text Word]) OR Capdepont Teeth[Text Word]) OR Capdepont Tooth[Text Word]) OR Dentin* dysplasia[Text Word]) OR Dentin Sialophos-phoprotein[Text Word]) OR Dentinogenesis Imperfecta[MeSH Terms] (2062)

2(((((((Osteogenesis Imperfecta[Text Word]) OR Brittle bone[Text Word]) OR Lobstein* Disease[Text Word]) OR Bruck syndrome*[Text Word]) OR fibrogenesis imperfecta ossium[Text Word]) OR osteopsathyrosis[Text Word]) OR Fragilitas Ossium[Text Word]) OR Osteogenesis Imper-fecta[MeSH Terms] (5937)

3 (((teeth[Text Word]) OR tooth[Text Word]) OR dentin*[Text Word]) OR abnormality, teeth[MeSH Terms] (241641)

4 (((bond[Text Word]) OR mutant[Text Word]) OR disorder[Text Word]) OR genotype[Text Word] (1454295)

5 1 and 3 (2061)

6 1 and 3 and 4 (255)

7 collagen[Text Word] (220087)

8 COL1A1[Text Word] (3308)

9 COL1A2[Text Word] (1365)

10 collagen fib*[Text Word] (21298)

11 ((collagen type 1[Text Word]) OR (collagen type one[Text Word])) OR (collagen type I[Text Word]) (21149)

12 ((collagen type 2[Text Word]) OR (collagen type two[Text Word])) OR (collagen type II[Text Word]) (6739)

13 ((collagen type 4[Text Word]) OR (collagen type four[Text Word])) OR (collagen type IV[Text Word]) (5534)

14 5 and 7 (356)

15 5 and 10 (33)

16 5 and 11 (158)

17 5 and 12 (6)

18 5 and 13 (1)

19 5 and 8 (72)

20 5 and 9 (56)

21 6 and 7 (76)

22 6 and 10 (7)

23 6 and 11 (36)

24 6 and 12 (1)

25 6 and 13 (0)

26 6 and 8 (32)

27 6 and 9 (32)

28 1 and 2 (278)

29 28 and 3 (78)

30 29 and 7 (126)

31 29 and 10 (12)

32 29 and 11 (53)

33 29 and 12 (1)

34 29 and 13 (0)

35 29 and 8 (50)

36 29 and 9 (53)
